# The evaluation of disease awareness, caregiver burden, and workday loss in caregivers of COPD patients

**DOI:** 10.3906/sag-2001-239

**Published:** 2021-01-01

**Authors:** Ayşe BAHA, Nurdan KÖKTÜRK, Burcu ÖZTÜRK, Elif ÖZARI YILDIRIM, İpek ÖZMEN, Alev GÜRGÜN, Ayşe Füsun TOPÇU, Eylem AKPINAR, Funda ELMAS, Hadice SELİMOĞLU ŞEN, Nalan OGAN, Yeşim ÖNDER, Ömer Tamer DOĞAN, Mehmet POLATLI, Züleyha BİNGÖL, Turhan ECE, Eda ÇELİK, Metin AKGÜN, Eylem Sercan ÖZGÜR, Sibel ATIŞ NAYCI, İrem ŞERİFOĞLU, Can ATEŞ

**Affiliations:** 1 Department of Pulmonary Medicine, Faculty of Medicine, Near East University, Mersin, Turkey; 2 Department of Pulmonary Medicine, Faculty of Medicine, Gazi University, Ankara, Turkey; 3 Department of Pulmonary Medicine, Süreyyapaşa Training and Research Hospital, İstanbul, Turkey; 4 Department of Pulmonary Medicine, Faculty of Medicine, Ege University, İzmir, Turkey; 5 Department of Pulmonary Medicine, Faculty of Medicine, Dicle University, Diyarbakır, Turkey; 6 Department of Pulmonary Medicine, Faculty of Medicine, Ufuk University, Ankara, Turkey; 7 Department of Pulmonary Medicine, Faculty of Medicine, Cumhuriyet University, Sivas, Turkey; 8 Department of Pulmonary Medicine, Faculty of Medicine, Adnan Menderes University, Aydın, Turkey; 9 Department of Pulmonary Medicine, Faculty of Medicine, İstanbul University, İstanbul, Turkey; 10 Department of Pulmonary Medicine, Faculty of Medicine, Atatürk University, Erzurum, Turkey; 11 Department of Pulmonary Medicine, Faculty of Medicine, Mersin University, Mersin, Turkey; 12 Department of Pulmonary Medicine, Ankara Atatürk Training and Research Hospital, Ankara, Turkey; 13 Department of Biostatistics, Faculty of Medicine, Yüzüncüyıl University, Van, Turkey

**Keywords:** Burden, caregiver, COPD

## Abstract

**Background/aim:**

Our aim is to determine the caregiver burden of chronic obstructive lung disease (COPD) patient’s caregivers, and to determine whether there is a workday loss.

**Materials and methods:**

252 COPD patients and their caregivers were included. Disease information of the patients were recorded and a questionnaire was applied. Socio-demographic characteristics of the caregivers were recorded and a questionnaire consisting of 24 questions including COPD disease, treatment and loss of working days, and the Zarit Scale were used.

**Results:**

128(50.8%) of the patients according to GOLD were group-D, 97(38.5%) of the patient’s relatives were working, 62(24.7%) were not able to go to work for 1–14 days, and 125(57.1%) spent outside the home from 1–14 nights, because those accompanied to patients. In univariate analysis were detected modified medical research council (mMRC) (p < 0.001), CAT (p < 0.001), the number of comorbidities of patients (p = 0.027), forced expiratory volume in 1 FEV1cc (p = 0.009), FEV1% (p < 0.001), the presence of long term oxygen therapy (LTOT), and the number of comorbidities of the patient’s relatives (p = 0.06) increased the care load. In multiple linear regression analysis, age (p = 0.03), COPD assessment test (CAT) score (p = 0.001), FEV1% (<0.068) and the number of comorbidities of patients (p = 0.01) and the number of comorbidities of caregivers (p = 0.003) increased the caregiving burden.

**Conclusion:**

In COPD increases caregiving burden. This burden is greater in symptomatic patients and when comorbidities are present. Psychosocial and legal regulations should be investigated and solutions should be produced for the caregivers of COPD patients.

## 1. Introduction

The duration of time together with chronic diseases is increasing all over the world, because of the aging of societies, newly developed drugs and developments in the field of medicine. This situation increases the burden of caregiver^1^. Caregiver burden is used to express the physical, psychological, social or financial reactions that can be experienced while providing care [1]. Furthermore, caregiver burden has been identified as a risk factor for worsening caregiver physical and psychological health, worsening health-related quality of life, compromised immunity, and mortality [2–4]. Determining the care burden levels of caregivers is important for increasing the quality of life of both caregivers and patients [5–7].

Chronic obstructive pulmonary disease (COPD) is an important global health problem associated with increased mortality and morbidity^2^. COPD causes symptoms such as dyspnea, cough, phlegm, loss of appetite, insomnia, fatigue, depression, anxiety, and insufficiency in cognitive functions [8]. All of these physical and psychosocial problems decrease the functional performance of individuals, cause limitations in their daily life activities, and increase both the dependence and care needs of individuals [9,10].

In Europe and the USA, 4%–10% of the adult population have COPD and its prevalence is increasing [11,12]. In the Burden of Obstructive Lung Disease (BOLD) study which is performed in Adana, 19% of adults over 40 years old are estimated to have COPD [13]. The burden of COPD on the health care system and society is increasing day by day [14]. There are little data on the burden of caregivers and the awareness of the disease in COPD, which is the third most common cause of death among chronic diseases [15]. Even, there is almost no data on the number of workday loss of informal caregivers of COPD patients and the number of nights spent outside home^2^.

The aim of the study that in caregivers of COPD patients to determine caregiver burden, the workday loss and the awareness of caregivers about COPD disease and treatment.

## 2. Materials and methods

This is a prospective, cross sectional, questionnaire study. Between September 1, 2017 and December 31, 2017, 252 patients with COPD and 252 their informal caregiver were included in the study, from 12 centers. The only inpatients and their primary caregivers who were included in the study were informed about the purpose of the study, their written and verbal consents were obtained, and they were made to sign the participant consent form. It was approved by Ethical Committee of Ufuk University Faculty of Medicine.

### 2.1. Definition of COPD

Patients with forced expiratory volume in 1 s (FEV1/FVC) less than 70% in the pulmonary function tests (PFTs) in the stable condition were diagnosed as COPD. Patients with COPD for at least for 6 months were included in the study. All patients were staged according to Global Obstructive Lung Disease (GOLD) 2017 combined assessment as GOLD stage A, B, C, and D^2^. Spirometric staging was not used in this new classification. Staging have been made according to the degree of symptoms, and the number of COPD exacerbations in the last 1 year. Accordingly, GOLD-A defines the patient who has the least symptoms (mMRC 0–1 or CAT<10) and 1 or less exacerbation in the last year. GOLD-B defines the patient who has more symptomatic (mMRC ≥2 or CAT≥10) and 1 or less exacerbation in the last year. GOLD-C defines the patient who has less symptomatic (mMRC 0–1 or CAT<10) and ≥2 exacerbation or ≥1 hospitalization in the last 1 year. GOLD-D defines the patient who has more symptomatic (mMRC ≥2 or CAT≥10) and ≥2 exacerbation or ≥1 hospitalization in the last 1 year.

### 2.2. Definition of caregivers

Caregivers were defined as family members who were involved in the patient’s daily care needs, such as; transport to the hospital, application of the treatment at home, personal care, and shopping.

### 2.3. Data collection

Data of questionnaires and socio-demographic information were collected by conducting face-to-face interviews with COPD patients and their caregivers.

### 2.4. Patient and caregiver information form

The demographic characteristics, disease information (GOLD grade and stage, the dyspne scale of modified medical research council (mMRC), COPD assessment test (CAT)), the number of comorbidities, treatment and disease conditions in the last 1 year (emergency service admissions, hospitalization, number of exacerbations) of the patients were recorded. The demographic characteristics, the number of co-morbidities, working conditions, educational levels, and the marital status of the caregivers were recorded. In addition, a questionnaire consisting of 12 questions related to disease awareness (5 questions), treatment awareness (5 questions) and work day loss (2 questions), and the Zarit Burden interview scale (see below) was applied.

### 2.5. Zarit burden interview (ZBI)

The scale (ZBI) was developed by Zarit et al. [18], and the study of Turkish validity and reliability was conducted by İnci and Erdem and the internal consistency coefficient of the scale was determined to be 0.92 [18]. The scale consists of 22 statements determining the effect of caregiving on the life of individuals. The ZBI measures subjective burden in terms of the degree (from ‘never: 0’ to ‘almost always: 4’) to which the caregiver experiences physical, psychological, emotional, social, and financial problems as a result of their care-giving role. As the score increases, the care burden increases and with the maximum score at 88 [18].

### 2.6. Statistical analysis

SPSS software (v: 22.0; SPSS Inc., Chicago, IL, USA), was used for statistical analyses. A p < 0.05 was considered statistically significant. In statistical analysis, categorical variables were given as numbers and percentages, and continuous variables were presented with mean ± standard deviation (SD) for descriptive analyses. Chi-square tests were used for comparison of categorical variables between the groups. In the comparison of categorical groups, if the percentage of the expected value less than 5 for each unit is greater than 20%, chi-square analysis was performed using the Monte Carlo method. In order to identify variables increasing the burden of care, multiple linear regression analysis was performed. The linear regression analyses data are presented as beta coefficient with p value. In the relationship between the dependent variable and the independent variables, when significant correlations in which greater than 0.70 were found between independent variables, only one of those variables was taken. For the control of multiple linearity, the VIF value below 5 was applied. Backward method was applied while creating the linear regression model. Variables with p < 0.05 indicated a significant association between the variables and the outcomes.

## 3. Results

Of the 252 patients, 221 (87.7%) were male and 31 (12.3%) were female and the mean age was 66.8 ± 9.7 years. Fifty-four (21.4%) patients were active smokers and 159 (63.1%) were exsmoker. Eighty-three (32.9%) patients had one, 45 (17.9%) patients had two, 28 (11.1%) patients had three and 10 (4%) had more than 3 comorbidities. Overall, 25.4% (n = 64) of the patients live in the countryside and 68.3% (n = 172) in urban cities. Disease characteristics related to COPD are shown in Table 1. Accordingly, approximately half of the patients (50.8%) were GOLD-D. Seventy-seven (30.6%) patients had long term oxygen therapy (LTOT), 42 (16.7%) had bi-level positive airway pressure (BPAP), and 123 (48.8%) had a nebulizer.

Sociodemographical characteristics and comorbidities of caregivers are shown Table 2. 41.3% (n = 104) of the caregivers were spouse, 42.8% (n = 108) were the child, and 15.9% (n = 40) were other relatives. Of the 252 caregivers, 167 (66.3%) were female and 85 (33.7%) were male. The mean age was 48.1 ± 13.4 years. Almost half of caregivers (n = 125, 49.6%) had never smoked, and 55 had biomass exposure. Eighty-two (32.5%) caregivers had at least one comorbidity. The number of comorbidity of the caregivers is as follows; 43 (17.1%) had 1 comorbidity, 17 (6.7%) had 2 comorbidities, 10 (4%) had 3 comorbidities, and 3 (1.2%) had ^3^3 comorbidities. 39.6% (n = 98) of caregivers were employee.

The questions that were asked to caregivers about COPD disease awareness, treatment awareness and loss of the working days are shown in Table 3. Two-hundred and thirty eight of the caregivers (94.4%) were aware of that COPD is a lung disease and 232 (92.1%) knew the most common cause of COPD is smoking. Only 52.2% (n = 131) of caregivers knew that COPD is a treatable disease. Sixty-two (46.3%) caregivers suffered at least one work day loss and 125 (53.5%) caregivers spent at least one night outside home because of their stay in the hospital.

Table 4 shows the number of lost working days due to accompanying COPD patients in the hospital. According to Table 4, as the severity of the COPD increases, the loss of working days of caregivers increases. Caregivers of GOLD D group patients have lost working days the most.

According to GOLD combined evaluation, Zarit total scores were 19.3 ± 10.1 (median = 18) in group A, 24.9 ± 15.1 (median = 22) in group B, 22.1 ± 13.9 (median = 19) in group C and 30.5 ± 15.6 (median = 29) in group D patients.

The factors that increase the burden of care in the univariate linear regression analysis were the age (<0.001), mMRC (p < 0.001), CAT (p < 0.001), the number of comorbidities of patients (p = 0.02), FEV1 (mL) (p = 0.009), FEV1% (p < 0.001), LTOT (p = 0.03), using a nebuliser (p = 0.002), and the presence of comorbidities of caregivers (p = 0.006). The factors that increase the burden of care in the multivariate linear regression analysis were age, CAT score, the number of comorbidities of patients, FEV1%, and the presence of comorbidities of caregivers (Table 5).

## 4. Discussion

The most important results of this study are that Zarit Scale of caregivers and workday losses increased as the severity of the COPD increases. Another result is that awareness of disease and treatment in caregivers is relatively high. This study is the first study in the literature for the evaluation of COPD disease-treatment awareness and it is the second study that assessed workday loss of caregivers of COPD patients [17].

Caregiver burden was investigated in many chronic diseases [18–21]. Due to the psychological, physical, and social difficulties they are exposed to, the caregivers of chronic patients have been described as “hidden patients” in the literature [22].

In the study Goris et al., mean scores of the care burden of caregivers were found to be 40.91 ± 20.58 [23]. Similarly, Tel et al. showed that mean scores of care burden of caregivers of patients with COPD were 39.64 ± 15.07 [24]. The literature involves different studies in which the care burden is higher (50.2 ± 8.7) as well as lower (22.80 ± 14.45) [25,26].

In a study in which 179 COPD and chronic heart failure patients were analyzed, only 10% of caregivers reported no burden [27]. In our study, the Zarit Burden Scale mean score were 19.3 ± 10.1 (median = 18) in group A and 30.5 ± 15.6 (median = 29) in group D patients. We did not find any other study that calculated the Zarit score in relation to COPD groups. In our study, Zarit score was lower in Group A and C, and was higher in Group B and D. That is, the caregiver burden was proportional to the severity of the dyspnea, but not with the spirometric severity. According to GOLD, COPD Group B, and D patients have higher dyspnea than Group A and C^2^. Therefore, we think that dyspnea increases caregiver burden.

The relationship between the age of patient and caregiver burden differs from disease to disease. In a study conducted by Goris et al. [23] including caregivers of patients with COPD, there was no significant relationship between the patient’s age and the caregiver burden. But in our study, there was correlation between age and caregiver burden. To date, age has been correlated with caregiver burden in many chronic diseases [28,29]. In the study of McNabney et al., age and COPD were found to be among the most important predictors of care commitment [30].

In another study, it was found that caregiver burden increased as age increased in oncology patients [31]. In our study, neither the age of patients nor the age of the caregiver was correlated with the caregiver burden. This difference may be due to the different patient populations, different caregiver populations, and different nations in the both studies.

In 2009, Adelman et al. reported that an estimated 65.7 million individuals in the United States served as unpaid family caregivers and of these, 43.5 million (66%) provided care for an adult older than 50 years [32]. Also the majority of caregivers were women who took care of a relative (86%) or friend (14%) [34]. In another study, Goris et al. showed that caregiver burden scores of female caregivers were higher than those of male caregivers [23]. Godoy-Ramirez et al. found that the large majority (76.8%) of severe/very severe COPD patients were living with a caregiver, generally a wife or daughter [33]. In a meta-analysis, it was stated that female caregivers had lower physical well-being and higher levels of care burden and depression, compared with male ones [33]. Our findings were similar to previous studies [23,33]. 66.3% of caregivers were female and mostly, they were daughters or sons (42.4%, n = 108) or spouses (41.3%, n = 104) of the patients.

Forty percent of the caregivers in our study were both paid workers and informal caregivers of COPD patients. These caregivers accompanied COPD patients during routine hospital visits and access to health services at the time of exacerbations. According to our study the workday loss of caregivers is the least in the GOLD A group and is the highest in the GOLD D group. In the Global Obstructive Lung Disease report, the patients with annual hospitalization number of 1 or more per year due to exacerbations are categorized as group C and D^2^. Therefore, workday loss is expected to be higher in the caregivers of group D patients. According to GOLD report, Group B patients have fewer hospitalization number but they are more symptomatic than Group C. However, the annual workday loss in Group B patients is higher than in Group C in our study. In this case, it would be wrong to evaluate the workday loss of caregivers according to the number of hospitalizations of COPD patients. Because hospitalization of the patients during exacerbation depends on many factors such as availability of access to health care centers, affordability of health expenses, hospital occupancy rate, and level of perception of the patient’s symptoms. To date, there are not enough studies evaluating the workday loss of caregivers of COPD patients.

In our study, FEV1% was found to be associated with care burden. However, there is no other study evaluating the relationship between FEV1 and caregiver burden. Therefore, we could not make a comparison.

In the GARD study (Turkey’s asthma and COPD awareness study) 8527 people from the general population were included and 49.6% said that COPD is a lung disease [35]. In a recent study in China, the COPD awareness rate was 9.2% [36]. However, these studies were conducted in the general population. To the best of our knowledge, there is no study evaluating the awareness of caregivers about COPD disease and its treatment. The level of knowledge of caregivers in chronic diseases is one of the factors influencing the prognosis of the patient [37]. Caregivers are the people who accompany the patient during the disease and its treatment course. Therefore, we think that the awareness of the caregivers will affect the care course. The results of our study show that caregivers have a relatively high awareness when compared with other studies, although there are limited data in the literature. Approximately 50% of our patients have advanced stage COPD and their severity of symptoms and rate of hospitalization are high in the last 1 year. Therefore, it can be considered that the patient and the caregiver have enough experience with the disease and its treatment course.

A major limitation of our study is the low number of patients and their caregivers. The main reason for the low number of patients was the short duration (3 months) of data gathering period. Here we publish not only the data about the caregivers of COPD patients but also data about treatment awareness of patients. Therefore, the questions asked to the patients and their relatives are much more than they appear in our manuscript. The time needed to fill out questionnaires was about 30 min per person so that the number of questions were limited. Another limitation of our study is that we did not evaluate the financial and moral losses associated with work losses.

As a result, the caregiver burden of the caregivers of COPD patients are high, and caregivers have relatively high awareness of disease. The factors increasing the burden of care are the number of comorbidity and the severity of dyspnea of patients. In addition, caregivers with paid employment experience a workday loss due to accompanying patient. The socioeconomic consequences of the workday loss of caregivers are unknown, but this is a worthwhile investigation. It is a fact that diseases cannot be managed only with drugs or medical methods, and the caregivers are an important part of management of diseases. We think that the socioeconomic problems of caregivers should be determined and supported.

## Figures and Tables

**Table 1:** Characteristics of disease of COPD patients.

Parameter		n (%)
COPD group	GOLD A	37 (14.7)
	GOLD B	61 (24.2)
	GOLD C	24 (9.5)
	GOLD D	128 (50.8)
FEV1 (mL/%) (mean ± SD)		1293 ± 622/48.1 ± 19.5
FVC (mL/%) (mean ± SD)		2182 ± 965/65.1 ± 19.5
FEV1/FVC (mean ± SD)		57.2 ± 10.6
CAT (mean ± SD)		18.94 ± 9.5

Abbreviation COPD; chronic obstructive lung disease, FEV1; forced expiratory volume in one second, FVC; forced vital capacity, CAT; COPD assessment test.

**Table 2:** Smoking status, working status, working hours, marital status, and educational level of caregivers.

Parameters		Caregivers
Smoking status (n-%)	Smoker	76 (30.2)
	Exsmoker	51 (20.2)
Smoking pack-year		20.6 ± 14.2
Working status	Unemployed	151 (60.4)
	Employee	98 (39.6)
Working hours	Full day	66 (73.3)
	Half day	9 (10)
	Shift	15 (16.7)
Marital status	Married	189 (75.6)
	Single	58 (23.2)
Educational level	Not literate	19 (7.6)
	Primary school	78 (31.3)
	Secondary school	33 (13.3)
	High school	61 (24.5)
	University	53 (21.3)
	Others	5 (2)

**Table 3:** The questions that were asked to caregivers about COPD disease awareness, treatment awareness and loss of the working days.

Questions	Yes, n (%)	No, n (%)	No idea, n (%)
1. COPD is a lung disease.	238 (94.4)	-	13 (5.2)
2. The most common cause of COPD is smoking	232 (92.1)	6 (2.4)	14(5.6)
3. Exposure to occupational dust and chemicals and indoor air pollution may result in COPD.	198 (78.9)	17 (6.8)	36 (14.3)
4. COPD is a treatable disease.	131 (52.2)	59 (23.5)	61 (24.3)
5. The reason for hospital admissions in COPD is not only lung problems, but also the severity of comorbidities.	138 (55.2)	41 (16.4)	71 (28.4)
6. Using inhalation therapy with the right technique reduces cough, sputum and shortness of breath.	206 (82.7)	3 (1.2)	40 (16.1)
7. Do you apply nondrug method to reduce cough, sputum, and dyspnea?	61 (24.3)	190 (75.7)	-
8. Using inhaled therapy with the right technique reduces the frequency of emergency department visits and the frequency of hospitalization.	172 (68.3)	20 (8)	58 (23.2)
9. Do you apply nondrug method to reduce the frequency of hospital admission and the frequency of emergency services?	34 (13.5)	217 (86.5)	-
10. Have you received training on behavior change in the treatment of COPD?	87 (34.7)	137 (54.6)	27 (10.8)
11. How many working days did you lose in the last year because of accompanying the patient in the hospital?	0 day	72 (53.7)	27 (10.8)
	1–7 days	36 (26.9)	
	8–14 days	17 (12.7)	
	>14 days	9 (6.7)	
12. How many nights have you spent outside the house in the last year because of accompanying the patient in the hospital?	0 day	108 (46.4)	
	1–7 days	73 (31.3)	
	8–14 days	23 (9.9)	
	>14 days	29 (12.4)	

**Table 4:** The number of lost working days in relation to the severity of COPD.

COPD group	0 n (%)	1–7 days n (%)	8–14 days n (%)	>14 days n (%)	Total n (%)	p
GOLD A	26 (74.3)	8 (22.9)	-	1 (2.9)	35 (100)	<0.001
GOLD B	37 (66.1)	8 (14.3)	8 (14.3)	3 (5.4)	56 (100)	
GOLD C	7 (33.3)	11 (52.4)	1 (4.8)	2 (9.5)	21 (100)	
GOLD D	37 (31.1)	45 (37.8)	14 (11.8)	23 (19.3)	119 (100)	

**Table 5:** The factors that increase the burden of care in the multiple linear regression analysis.

	Unstandardized coefficients		Standardized coefficients	t	P	95.0% Confidence interval for B	
	B	Std. error	Beta			Lower bound	Upper bound
Age	−0.238	0.109	−0.136	−2.187	0.03	−0.453	−0.023
CAT score	0.393	0.107	0.241	3.677	<0.001	0.182	0.604
The number of comorbidities	3.571	0.817	0.273	4.373	<0.001	1.961	5.18
FEV1%	−0.092	0.05	−0.119	−1.833	0.068	−0.19	0.007
The number of comorbidities of caregivers	5.975	1.953	0.186	3.059	0.003	2.125	9.824
(Constant)	33.337	8.371		3.982	<0.001	16.834	49.839

Abbreviations: CAT; COPD assessment test.

## Footnotes

1 World Health Organization. Ethical choices in long-term care: what does justice require? Geneva, World Health Organization, 2002. Accessed on 23 March 2021.

2 Global Initiative for Chronic Obstructive Lung Disease (GOLD). Global strategy for the diagnosis, management and prevention of chronic obstructive lung disease. 2014. http://www.goldcopd.org. Accessed 23 March 2021.
